# Evidence-based decision-making for diagnostic and therapeutic methods: the changing landscape of assessment approaches in Germany

**DOI:** 10.1186/s12961-017-0253-1

**Published:** 2017-10-17

**Authors:** Britta Olberg, Sabine Fuchs, Katja Matthias, Alexandra Nolting, Matthias Perleth, Reinhard Busse

**Affiliations:** 1 Berlin University of Technology, Straße des 17. Juni 135, 10623 Berlin, Germany; 2 Federal Joint Committee, Wegelystraße 8, 10623 Berlin, Germany

## Abstract

This article examines the current status and most important changes over time to the legislative framework on the health technology assessment-informed decision-making process on diagnostic and therapeutic ‘methods’ in Germany. The relevant information was obtained through documentary analysis covering the period 1990 to 2017. The findings show that, even if the outpatient care sector appears to be much more regulated than the inpatient sector (based on a strict separation of the two care settings), developments in Germany have led to a more tightened assessment framework, making the use of evidence a firm component in the decision-making process. Nevertheless, a comprehensive approach for a systematic assessment of diagnostic and therapeutic ‘methods’ still does not exist. Readjustments of current regulations in Germany, such as the existing ‘*Verbotsvorbehalt*’ (i.e. provision of a diagnostic and therapeutic ‘method’ possible unless actively delisted) in the inpatient care setting, as well as further developments at the European level are needed in order to create a system that ensures early access to innovation under controlled study conditions.

## Background

The debate on decision-making in healthcare informed by evidence (health technology assessment; HTA) is not a recent phenomenon. Many European countries have introduced national or regional technology assessment programmes during the past three decades [1–9], mainly triggered by growing concerns over the benefit/value of innovations in terms of outcomes relevant for patients, safety and budget impact. The discussion about licensing of medical devices (MDs) in Europe may serve as an example. Complications with metal-on-metal hip implants pointed towards systemic shortcomings of the current regulation of MDs, which poses a clear threat to patient safety. The widely held view that the system for MD regulation in Europe is flawed, especially regarding the regulatory requirements for clinical data on MDs, triggered not only a stricter MD regulation at the European Union (EU) level (e.g. the revision of the EU directives) [10], but also stimulated activities at the national level, as for example in Germany. This becomes evident when considering legislative changes over past years that comprise a more tightened decision-making framework on hospital-based diagnostic and therapeutic methods (‘*Untersuchungs- und Behandlungsmethoden*’; more details regarding the definition are given in the methods section) employing high-risk MDs, making the use of HTA an essential aspect in the decision-making process. However, besides the increased activity of HTA in Europe and worldwide and the common remits, roles and aims national healthcare systems share, there are differences in terms of its implementation and impact [1, 2].

For example, within the corporatist German system, with its multitude of actors in the so-called self-governance or self-administration, represented by the Federal Joint Committee (*Gemeinsamer Bundesausschuss*, G-BA), the process for HTA markedly differs to those in most other countries [11]. In self-governance, payers and providers are mandated to ensure access to and provision of health services [12]. This includes the assessment process of diagnostic and therapeutic methods, on the one hand, and the final decision by the same actors with sometimes conflicting interests in these processes, on the other. Moreover, different regulations for diagnostic and therapeutic methods between the in- and outpatient care setting in Germany exist. Whereas in outpatient care any new diagnostic and therapeutic method must be evaluated before being reimbursed (referred to as ‘*Erlaubnisvorbehalt*’ regulated under §135 of the Social Code Book Five; SGB V), in inpatient care new diagnostic and therapeutic methods are reimbursed without prior assessment, as long as fundamental principles of quality and cost-effectiveness are not violated and methods are not delisted by the G-BA (referred to as ‘*Verbotsvorbehalt*’, regulated under §137c SGB V).

Based on this and prompted by the current renewal of long-standing debates about the permissiveness of the German system compared to most other countries in terms of coverage of innovative yet unproven technologies, this study aims to examine the current status of evidence-based assessment in the context of diagnostic and therapeutic methods in Germany. Specifically, we focus on the role of HTA in decisions on health service coverage. This includes an analysis of the most important changes to the legislative framework over time, the dynamics that have informed them and their implications for the decision-maker.

## Methods

This analysis is informed by documentary analysis from 1990 to 2017 on the important reforms and regulations, focussing exclusively on evaluations/decisions of diagnostic and therapeutic methods.

The term ‘method’ has been evolving through several legislative decisions over the past 20 years. It is currently defined as a medical procedure as part of a physician-led treatment concept and is characterised by a certain degree of complexity, usually involving several steps, which may include the use of MDs. Moreover, there has to be an underlying theoretical and scientific concept, which differentiates the method from others (e.g. pharmaceuticals or one-step procedures) and thus justifies its systematic application in the diagnosis and treatment of specific diseases [13]. Consequently, this understanding of a procedure encompasses a broad spectrum of new diagnostic and therapeutic methods and entails a broader conception than the application of MDs alone [14].

Documents included were legal texts (e.g. records of court decisions), final scientific reports of coverage decisions after benefit assessments (including related justifications/reasons) by the Institute for Quality and Efficiency in Health Care (*Institut für Qualität und Wirtschaftlichkeit im Gesundheitswesen*; IQWiG) or other research institutes commissioned to conduct evidence reviews, selected publications on diagnostic and therapeutic methods, and materials from (governmental) websites or digital databases and/or archives (e.g. juris database; G-BA’s document management system) such as policy documents relating to the assessment of diagnostic and therapeutic methods published by the G-BA and by corporatist organisations.

## HTA in the German decision-making process for diagnostic and therapeutic methods – early development and the actors involved

HTA is defined as “*any process of examining and reporting properties of a medical technology used in healthcare, such as safety, efficacy, feasibility, and indications for use, cost, and cost-effectiveness, as well as social, economic, and ethical consequences, whether intended or unintended*” [15]. In Germany, HTA has been under discussion since the mid-1990s, closely related to the rise of evidence-based medicine (EbM). The principles and methods of HTA were continuously adopted by various decision-making bodies in Germany, such as the Federal Standing Committee of Physicians and Sickness Funds, the Hospital Committee and the coordination committee [16]. In 2004, within the Statutory Health Insurance (SHI) Modernisation Act, these committees were transformed into the G-BA. The basis for the work of the G-BA is the German SGB V, which defines the health policy framework in which the G-BA operates. This legislative framework is set by both chambers of parliament. The G-BA is the highest decision-making body of the joint self-government of physicians, dentists, hospitals and health insurance funds in Germany. Associations of office-based physicians (*Kassenärztliche Bundesvereinigung* (Federal Association of SHI Physicians)), dentists (*Kassenzahnärztliche Bundesvereinigung* (Federal Association of SHI Dentists)), hospitals (*Deutsche Krankenhausgesellschaft* (German Hospital Federation)), sickness funds (*GKV-Spitzenverband* (Federal Association of Sickness Funds)) and patients’ representatives are embodied in the G-BA’s decision-making committees [12]. The G-BA issues directives and thus determines the details of the statutory regulations. To inform the G-BA’s coverage decisions by an independent evidence assessment, the legislator in parallel also created the IQWiG. Its main task is to produce HTA reports on, for example, pharmaceuticals or diagnostic and therapeutic methods [17, 18]. While the establishment of IQWiG illustrates the separation of addressing the evidence within a standardised process of providing an HTA report to the G-BA, and the (coverage) decision-making process within the G-BA, in practice there is interaction between these processes. The G-BA does not necessarily follow the recommendations issued by IQWiG or requests additional evidence either from IQWiG or from other sources.

A harmonisation of the basic rules set by the legislator of the two sectors (*Verbotsvorbehalt* and *Erlaubnisvorbehalt*) was not established, so the same evidence can lead to different decisions. In addition, the G-BA was not authorised to initiate studies in situations where the evidence body was thought to be insufficient. To understand the current situation in Germany, it is therefore important to be familiar with further regulations set from 2012 onwards.

The following two sections describe the processes prior to 2012 and from 2012 onwards in detail. As already indicated above, different regulations for the assessment of diagnostic and therapeutic methods between the in- and outpatient setting in Germany exist. Therefore, the two sectors need to be addressed separately with respect to their impact on assessment approaches within the context of evidence-based decision-making.

## Assessment approaches of diagnostic and therapeutic methods prior to 2012

The use of scientific evidence plays one key role in the decisions of the G-BA. This becomes clear by the fact that it is embedded in the G-BAs’ rules of procedure (*Verfahrensordnung*), which are founded on the international principles of EbM. Applying these principles means integrating the best available scientific evidence with clinical expertise and patient values [19]. Moreover, the rules of procedure make specific stipulations for decision on the coverage of new diagnostic and therapeutic methods, stating that they have to demonstrate evidence of the “*accepted state of medical knowledge about the benefits, necessity and efficiency*”; methods that are “*not necessary or inefficient*” should not be included in the SHI benefit basket [20]. However, as to what constitutes necessity is sometimes controversial between the actors in the G-BA.

### Assessment approaches of diagnostic and therapeutic methods in the outpatient sector

#### Period from 1990 to 1997

**Fig. 1:**
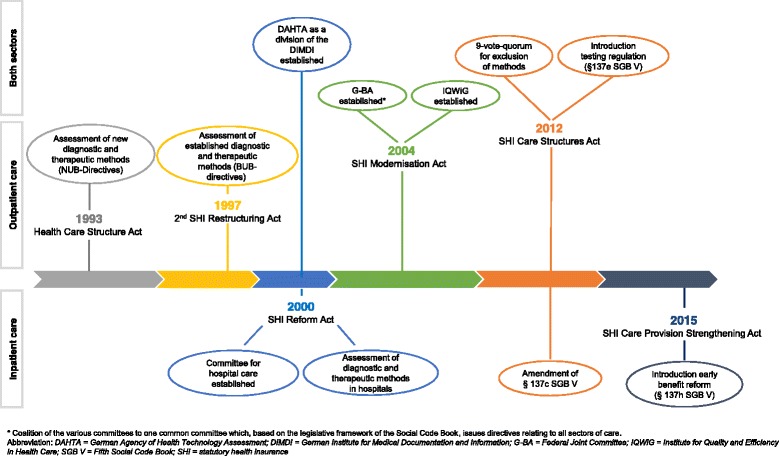
Regulatory milestones regarding the assessment of diagnostic and therapeutic methods in Germany (simplified illustration)

In the 1990s, the Federal Standing Committee of Physicians and Sickness Funds (the predecessor of the G-BA) established the evidence-based assessment of diagnostic and therapeutic methods in the outpatient care setting [21]. This was triggered by rising criticism that decisions were intransparent and essentially based on statements by purely relying on the opinion of individual experts rather than relying on critical appraisal of scientific evidence available. Concerns had been raised regarding the implementation of a more transparent and reliable evidence-based assessment approach. As a reaction, the Federal Standing Committee of Physicians and Sickness Funds incorporated the evidence-based selection and assessment of the scientific literature as a basis for decisions regarding the inclusion or exclusion of diagnostic and therapeutic methods in the SHI benefit basket. Furthermore, resulting from the transition to the so-called NUB-directives, which is the acronym for ‘*Neue Untersuchungs- und Behandlungsmethoden*’, the classification of scientific studies with different trial designs (and varying certainty of the results) became a legal component for the assessment process [21, 22] (Fig. 1). The ambulatory execution of low-density lipoprotein elimination as an extracorporeal apheresis was the first accepted diagnostic and therapeutic method under these newly introduced NUB-directives. For seven methods, such as the Heidelberg capsule, electro acupuncture according to Voll or the immune-augmentative therapy, no therapeutic and/or diagnostic benefit was approved by the committee. Therefore, these methods were not included in the SHI benefit basket [23].

#### Period from 1998 to 2011

Despite these developments until 1997, the decision-making process of the Federal Standing Committee of Physicians and Sickness Funds was less transparent, as only the decisions themselves were published but not how they were formed (i.e. through published rules of procedure). Its work was being increasingly denounced as arbitrary and incoherent [24]. In the context of this criticism, the Federal Association of SHI Physicians, together with representatives of the sickness funds, developed a new mode of operation as of January 1, 1998. This led to the implementation of a procedural guideline for evidence-based assessment in the subcommittee on medical treatment (within the second SHI Restructuring Act in 1997), which introduced further substantial changes of the regulatory framework, especially improving the transparency of the decision-making process. Specifically, this included, for example, (1) publication of the process for assessment, (2) publication of the consultancy topic with a general call for written comments and (3) publication of a comprehensive final report including a presentation of the comments, the scientific literature, the entire consultation process and the consultancy results [17, 21, 25]. In addition, this reform extended the Committee’s assessment responsibilities to the assessment of already established methods (Fig. 1). In November 1997, the Federal Standing Committee of Physicians and Sickness Funds published its first announcement listing two established technologies for re-assessment, i.e. bone densitometry measurement and methadone substitution, and six new technologies for evaluation. A second announcement in June 1998 listed an additional seven new technologies for assessment [22]. The final decision regarding the bone densitometry measurement in 1999 represents an important example for informed decision-making using HTA. In addition to written statements, guidelines, and an analysis of the scientific literature, a HTA report [26], commissioned by the Federal Ministry of Health, was considered during the decision-making process. Based on all findings, bone densitometry measurement was the first method issued with a restriction to a specific indication due to a lack of evidence for the remaining indications.

Prior to 2012, it was possible to suspend benefit assessments for methods, if valid studies were expected to be finalised and/or published in the near future. However, there was no possibility to commission evaluation, such as the initiation of a clinical trial, if study evidence was missing (Table 1). This was only possible as pilot projects within separate contracts between sickness funds and physicians. One example is the decision on acupuncture. In this case, the G-BA concluded that evidence for efficacy, safety and effectiveness was not sufficient to decide on SHI coverage, but that a comprehensive assessment of these factors in relation to chronic low back pain, chronic headache and chronic painful arthritis of large joints was required. Consequently, many sickness funds consecutively launched three major acupuncture pilot projects, including randomised controlled trials, to assess these three indications.

**Table 1:** Assessment approaches of the G-BA and resulting consequences for provision prior to and after 2012

	Outpatient		Inpatient		
	*Until 2011*	*Since 2012* ^*a),b)*^	*2000-2011*	*2012-2016* ^*a),b)*^	*Since 2017*
Before evaluation	Not in benefit basket, i.e. provision not reimbursable	Not in benefit basket, i.e. provision not reimbursable	Provision allowed	Provision allowed	Provision allowed, unless method under § 137h
After evaluation					
Proven benefit	Acknowledg-ment and inclusion in benefit basket	Acknowledg-ment and inclusion in benefit basket	Continued provision	Continued provision	Continued provision/ inclusion in benefit basket (§ 137h)
No proven benefit + no ‘potential’	No acknowledge-ment, i.e. non-inclusion into benefit basket	No acknowledge-ment, i.e. non-inclusion into benefit basket	Exclusion from benefit basket	Exclusion from benefit basket	Exclusion/ non-inclusion (§ 137h)
No proven benefit, but clinical studies expected (= ‘potential’)	Suspension of the decision until studies available	Suspension of the decision until studies available	Suspension of the decision until studies available	Suspension of the decision until studies available	Trial implementation (under § 137e)
Insufficient evidence, indication of possible benefit, no clinical studies expected/ planned (= ‘potential’)	Approach not existent	Trial implementation (under § 137e)	Approach not existent	Trial implementation (under § 137e)	Trial implementation (under § 137e)

^a^) The new regulations since 2012 complement the existing directives in the in- and outpatient care setting (§§ 135 and 137c SGB V)

^b)^ The term ’potential’ was introduced in 2012 in conjunction with the regulation under § 137e SGB V. This new assessment category requires a method to show potential being a valid diagnostic or treatment alternative to warrant the implementation of a trial. Additionally, the presence of a ’potential’ inhibits the exclusion of a method

### Assessment approaches of diagnostic and therapeutic methods in the inpatient sector

Most new diagnostic and therapeutic methods in Germany are first introduced in the inpatient sector, which is based on the principle referred to as *Verbotsvorbehalt* (regulated under §137c SGB V). This means that the provision of all diagnostic and therapeutic methods, including new ones, is allowed without any assessment of their benefit. In consequence, the G-BA has to decide only on the restriction or exclusion of already applied methods [14]. The primary intention by the legislator of this rule is to ensure that insured patients are rapidly able to take advantage of innovative methods of treatment. Therefore, in the inpatient sector, innovations in diagnostic and therapeutic methods find conditions that enable their rapid application in clinical practice [27]. However, the *Verbotsvorbehalt* also creates fundamental disincentives, as it releases manufacturers and hospitals from the responsibility to carry out valid studies needed to assess benefit and safety [28].

#### Period prior to 2012

Prior to 2000, the introduction of diagnostic and therapeutic methods was managed by individual hospitals in the context of budget negotiations with sickness funds or of applications for capital investment from the states [29].

In 2000, the SHI Reform Act was introduced. Based on this new legislation, a committee was established to assess whether a method sufficiently complies with the quality requirements defined by the SGB V [30]. Assessments have to be made in accordance with the processes established in the outpatient care setting, making the application of the principles of EbM also mandatory in the inpatient care sector (Fig. 1). The first indications announced for assessment under this new reform were autologous chondrocyte implantation, hyperbaric oxygen therapy and proton beam therapy [31]. However, under §137c SGB V, assessments in the inpatient setting can only be initiated by the request of representative stakeholders in cases where, for example, an indication of a negative risk-benefit ratio of a method exists. Consequently, in most cases, a benefit assessment occurs at a point in time when methods have already been established in the healthcare system for many years, despite considerable doubts as to whether their application sufficiently meets the quality requirements [28]. This means that, by the end of 2011, the G-BA was only able to exclude methods in few cases if their benefit was not proven. Still, a systematic assessment of the methods’ benefit (or harm) was not a fixed component under the regulation based on §137c SGB V (Table 1).

## Assessment approaches of diagnostic and therapeutic methods from 2012 onwards

The developments described in the previous sections, especially the formation of the G-BA and IQWiG in 2004, reflect that the use of scientific evidence has become more relevant in decision-making over time. However, as in prior to 2012, still only a fraction of all innovations have been assessed since 2012, and differently in the in- and outpatient care sector. This can be explained by remaining inconsistencies between the two healthcare sectors in Germany [32]. Such inconsistencies constitute barriers for a successful implementation of assessment approaches within the scope of coverage decisions. Additional regulations outlined in the following sections have been, and are still being, introduced aiming at eliminating these inconsistencies.

### Introduction of the coverage with evidence development (CED) reform (§137e SGB V)

**Fig. 2:**
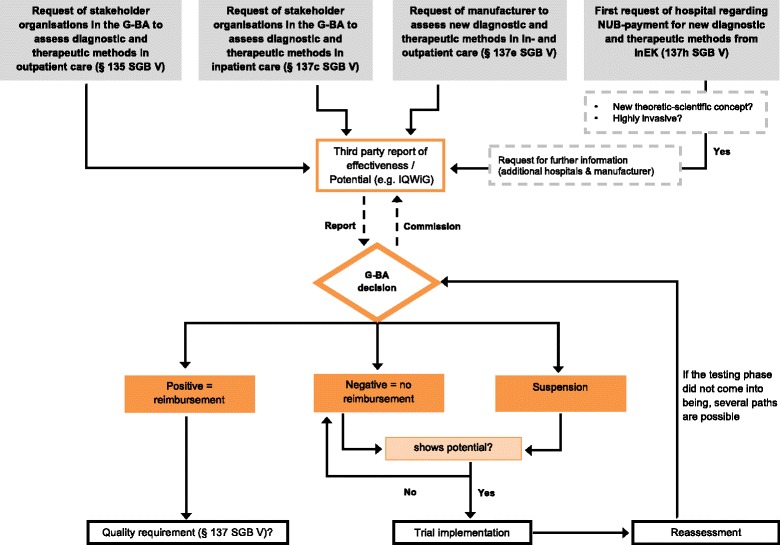
Assessment pathway of diagnostic and therapeutic methods in Germany (simplified)

Health policy makers have partially responded to the criticism from, for example, scientific associations, researchers and expert networks of the *Verbotsvorbehalt* in the inpatient care setting by introducing (within the SHI Care Structures Act in 2012) the testing regulation (Fig. 1), which can be considered a variety of the CED reform [14, 33]. This newly introduced stipulation (regulated under §137e SGB V) complements the existing regulations in the in- and outpatient care setting (§135 and §137c SGB V) (Fig. 2).

Consequently, the G-BA is not only able to assess the evidence, but also to initiate high-quality clinical studies. A key element when assessing a new method under the testing regulation is the determination of the ‘potential’ (i.e. promise of benefit) for being a valid diagnostic or treatment alternative, which is carried out by the IQWiG [34]. The potential is assessed against the mechanism of action of the new technology and the evidence available. Particularly, the method should likely be more effective, less complicated, less invasive, less harmful, or able to replace less efficacious methods in specific groups of patients [14]. This determination precedes each decision on testing according to §137e SGB V, which requires the presence of sufficient potential to warrant the generation of scientific evidence for a subsequent benefit assessment in a new study [27].

In cases where the G-BA – on the basis of the IQWiG’s results – assesses the method as showing potential and decides to conduct a trial, it develops and publishes a so called ‘testing directive’, which defines a trial phase for the method in question and commissions an independent scientific institute to plan and conduct the evaluation (Table 1) [14, 27]. Nevertheless, only few decisions have been published since the introduction of the testing regulation and no trial in the context of the testing directive has been conducted yet (reasons include difficulties regarding financing, the complex application process or the voluntary nature of this tool) (Table 2). Moreover, criticisms regarding the lack of transparency (e.g. lack of public information regarding new applications, no publication of negative G-BA votes on methods) as well as the long duration of the decision process remain. These aspects are of importance with respect to patient safety as, even if the implementation of a testing directive is initiated, the method in question remains available in the inpatient care setting (i.e. under §137c SGB V) without definitive knowledge about its benefit or harm for the patient.

**Table 2:** Methods fulfilling the criteria of the testing regulation (§137e SGB V) [49]

Year	Introduction of diagnostic and therapeutic methods according to the testing regulation (§137e SGB V)
2014	Non-invasive prenatal diagnosis to determine the risk of fetal trisomy 21 by means of molecular genetic testing
	Hyperbaric oxygen therapy during sudden hearing loss
	Measurement of fractionated exhaled nitric oxide for the detection of eosinophilic airway inflammation
	Measurement of fractionated exhaled monoxide for the control of asthma treatment during pregnancy
2015	Electric stimulation for tissue defect treatment in venous leg ulcers
	Electric stimulation for tissue defect treatment in diabetic foot ulcers
	Transcorneal electric stimulation in retinopathy pigmentosa
	Magnetic resonance tomography-controlled highly-focused ultrasound therapy for the treatment of the uterine myoma
2016	PET/CT in malignant lymphomas
	Measurement and monitoring of the pulmonary arterial pressure by an implanted sensor for the therapy optimisation in cardiac insufficiency in stage NYHA III
2017	Ultrasound-guided high-intensity-focused ultrasound for the treatment of uterine leiomyomas
	Ultrasound-guided high-intensity-focused ultrasound for the treatment of non-surgically treatable hepatocellular carcinoma

*CT* computed tomography, *PET* positron emission tomography, *NYHA* New York Heart Association, *SGB V* Fifth Social Code Book

Based on the same law, the legislator amended the voting rules in the G-BA and consequently increased the hurdle for the exclusion of a method. This is evident firstly in the introduced requirement of the two-thirds majority for such a decision, whereas a simple majority in the decision-making body still suffices for inclusion of a new method. Secondly, the increase in the hurdle is shown even more clearly by an amendment of §137c SGB V, which states that:

1. a method can only be excluded directly if the G-BA explicitly ascertains the ineffectiveness or even harm of the method, and
2. if the benefit of a method has not yet been adequately proven, but offers the potential of being a treatment alternative, the G-BA decides to issue a testing directive under §137e SGB V (Fig. 1) [20].

Accordingly, as stated by the former impartial chairman of the G-BA, Rainer Hess, methods can no longer be excluded directly, but only if they fall under the testing directive, are consequently evaluated, and show no potential to benefit or cause harm [35]. This requirement represents a reversal of the burden of proof and thus constitutes a retrograde step. It stands in clear contrast to the regulations concerning pharmaceuticals, for which a proof of the efficacy and safety has to be provided a priori within the framework of the market authorisation process [27]. Thus, the quality requirements defined by the SGB V in the inpatient care setting apply only to a limited extent, as ‘potential’ does not automatically mean that the method has any (proven) benefit or is free of harm. This involves the risk that, the longer innovations are being used outside the actually required studies, i.e. the more they become established in routine care, generation of valid clinical data on effectiveness and/or safety becomes less likely. Moreover, examples in the past (e.g. the metal-on-metal hip implant scandal and experiences like stents for the treatment of intracranial stenosis) have shown that innovations often can be ineffective or even harmful. In order to protect patients from such potentially harmful interventions, the German legislature aimed at correcting the deficiencies of the existing regulations by introducing a mandatory benefit assessment of diagnostic and therapeutic methods based on high-risk MDs [27, 32, 36]. The details of this new regulation will be outlined in the following section.

### The early benefit assessment reform (§137h SGB V)

A new stipulation, based on the SHI Care Provision Strengthening Act in 2015, has been recently set in place (Fig. 1). This new directive (referred to as §137h SGB V) amends the existing regulations under §137c and §137e SGB V (Fig. 2) and further extends the possibilities of the G-BA with respect to the assessment of new diagnostic and therapeutic methods in the inpatient care setting. More precisely, this new directive commissions the G-BA to perform an early benefit assessment regarding new diagnostic and therapeutic methods based on high-risk MDs. According to the legal provisions, new diagnostic and therapeutic methods that are based on a ‘new theoretical and scientific concept’ and involve the use of highly-invasive class IIb, III or active implantable MDs are subject to the new assessment regulation. Originally, the Government Coalition Agreement of 2013 stated that “*hospitals that use new high-risk medical products will be obliged to participate in benefit and safety studies of the G-BA during the post-market launch phase*” [36]. These would be, according to the valid definition of MDs, all devices of risk classes IIb and III. However, after passing through Parliament and the Federal Ministry of Health, only ‘highly invasive’ MDs meeting the criteria mentioned above remained subject to the new assessment regulation [28, 36]. Consequently, only a fraction of what was actually envisioned in the coalition agreement will be subject to the early benefit assessment by the G-BA [37]. Specifically, as published by the G-BA on March 17, 2017 [38], the number of eligible diagnostic and therapeutic methods currently amounts to only eight.

## Discussion

Much research has been undertaken to date describing and comparing HTA activities across organisations and countries, documenting a general increase in HTA utilisation in Europe and worldwide [1, 2, 39–42]. Moreover, these studies also show a considerable variety in HTA processes as well as the methods for evaluation that are dependent on, for example, the nature of the issue, the types, quality and quantity of the available studies, and the degree to which the issue affects stakeholders’ interests [43]. This variety reflects the diversity of healthcare and policy systems with their different mandates, coverage-decision mechanisms and roles of key actors in the decision-making process [44]. Within this context, Germany, with its unique self-administration system, can serve as a valuable example regarding coverage decisions on health services. The complexity of this self-administration system, with its multitude of actors in the decision-making process (i.e. corporatist system), becomes even more intense due to the different existing pathways regarding the regulatory mechanisms for diagnostic and therapeutic methods. Therefore, this study aimed at exploring the current status of the decision-making process regarding diagnostic and therapeutic methods in Germany. We specifically analysed the most important changes to the legislative framework over the last 20 years, focussing on the role of scientific evidence (HTA) in informing those decisions.

Our analysis shows that HTA-informed decision-making on diagnostic and therapeutic methods in Germany was not a major issue until 1997. However, from 1998 onward, and in particular after 2012, the importance of evidence utilisation (HTA) in legitimising decisions, despite some conflicting steps (e.g. the two-third majority for the exclusion of a method), increased overall. These developments took place regardless of the respective government and were especially geared towards the inpatient care setting, while the outpatient care setting appeared to be much more regulated right from the beginning of technology assessment. Prior to the German self-administration, the uptake of HTA-informed decision-making was met with hope from the payer’s side for more control of (promising, yet expensive) unproven innovations, but provoked resistance from hospital organisations who were afraid of not previously existent restrictions in service provision. In addition, since in- and outpatient carers are also competitors for many treatment options, HTA-informed decision-making interferes with conflicting interests of decision-makers in several ways.

### Shaping the framework for HTA-informed decision-making on diagnostic and therapeutic methods prior to 2012

The introduction of a procedural guideline for an evidence-based assessment of new diagnostic and therapeutic methods by the Federal Standing Committee of Physicians and Sickness Funds in 1998 can be interpreted as the first milestone in which scientific evidence use has become institutionally embedded in the decision process of health policy in Germany. In 1997, the tasks of the G-BA were extended from the exclusive assessment of new methods to an assessment of already established ones in the outpatient care setting. This foresighted decision and a number of further key legislative changes, including the introduction of a coordinating committee for technology assessment in the ambulatory and hospital sector and the establishment of HTA programmes, especially the IQWiG in 2004, continuously extended and increasingly converted the evaluation of diagnostic and therapeutic methods into an evidence-based assessment process until the end of 2011. However, despite the relevance of HTA having increased, our analysis shows that, since then, its impact could have been higher. Specifically, until the end of 2011, the G-BA could only exclude (or suspend) methods in case of proven harm or lacking evidence regarding patient benefit, but had no tools to commission evaluations in the case of missing evidence [18].

### Further advancements regarding HTA-informed decision-making on diagnostic and therapeutic methods from 2012 onwards

To address the above mentioned shortcoming, the CED (regulated under §137e SGB V) model established in 2012 further expanded the legal framework of the G-BA regarding the generation of evidence. However, even if under this approach a study for the assessment of the method in question is initiated, the method can still be provided in the inpatient care setting until a decision based on the completed trial has been taken. Moreover, subsequent regulations, such as the introduction of the two-third majority for the exclusion of a method as well as the establishment of the concept of a ‘potential’, considerably increased the hurdles for an exclusion of a method [45, 46]. This is alarming, as patients might be exposed to a potentially harmful method. To avoid repetition of problems such as those experienced with intracranial stents, the German Parliament recently decided on an extension in the law introducing a new rule (§137h SGB V) in which new diagnostic and therapeutic methods based on a high-risk MD must undergo a formal HTA process prior to being covered by the healthcare system [36, 37]. Based on this, the controversial principle of the *Verbotsvorbehalt* (in accordance with §137c SGB V) in hospitals was partly replaced by these processes. Aside from the recent developments at the European level regarding the stricter regulation of MDs, the current legislative change in Germany, namely setting a HTA of high-risk diagnostic and therapeutic methods, may ensure the necessary degree of safety of a treatment in the future at the national level.

### The G-BAs future role among other European assessment bodies

The institutionalisation of HTA in different European countries began in the late 1980s. Sweden established one of the first HTA agencies in 1987. Various national HTA programmes followed and further shaped the concept of HTA in the 1990s [47]. Among these programmes, the United Kingdom’s National Institute for Health and Care Excellence (NICE) is one of Europe’s largest and longest established assessment bodies. It might be possible that the United Kingdom’s decision to leave the EU will have consequences for NICE’s future contributions to the European regulatory and HTA networks such as EUnetHTA (e.g. assessments, partially or completely independent and different from the European consensus). Although the details will have to be determined over the coming years, other regulators and HTA bodies in the EU, such as the G-BA (strong regulator) and the IQWiG (methodological leadership), might assume more responsibility in a post-Brexit EU, especially considering that the German market for MDs is the largest in Europe [48].

### Strengths and limitations

The strength of this work lies in its extensive documentary analysis between 1990 and 2017. To our knowledge, this is the first study to examine the current status and most important legislative changes over time regarding the HTA-informed decision-making process of diagnostic and therapeutic methods in Germany. However, we acknowledge some limitations. Firstly, documentary analysis is a useful research tool with considerable merit as a methodology for policy evaluation and reform. However, future research might extend this analysis by other methodological approaches (e.g. validation by an interview survey). Secondly, focus on the decision-making process of diagnostic and therapeutic methods does not allow any conclusions for other health policy areas such as the regulation of pharmaceuticals.

## Conclusion

This analysis shows that, over the last 20 years, healthcare politics in Germany have been characterised by a large number of reforms that led to the development and application of new procedures. This led to a continuous extension of the regulatory assessment approaches of the G-BA, making evidence-based assessment an indispensable feature of the SHI system. However, even with the most recently introduced benefit assessment for certain diagnostic and therapeutic methods, including high-risk MDs, the legislator failed to create a comprehensive approach for a systematic assessment of diagnostic and therapeutic methods in Germany. Therefore, these developments should be interpreted as a ‘learning system’, in which certain issues still remain to be resolved (e.g. discrepant approaches to coverage decisions between ambulatory and hospital care).

To overcome the uncertainty regarding the effectiveness and safety of a method, particularly in the inpatient sector, a regulatory framework is needed to protect patients from ineffective or even harmful methods. This implies mandatory assessments of new methods conducted within acceptable deadlines in order to realise both a fast access to diagnostic and therapeutic methods for patients and planning security for manufacturers. The realisation of this approach does imply a realignment of the current regulations in Germany as well as further actions at the European level, helping to contain the risks associated with access to technologies without a robust evidence base.

## Abbreviations

CED: coverage with evidence development
G-BA: Gemeinsamer Bundesausschuss (Federal Joint Committee)
EBM: evidence-based medicine
EU: European Union
HTA: health technology assessment
IQWiG: Institut für Qualität und Wirtschaftlichkeit im Gesundheitswesen (Institute for Quality and Efficiency in Health Care)
MD: medical device
NUB: Neue Untersuchungs- und Behandlungsmethoden (new diagnostic and therapeutic methods)
NYHA: New York Heart Association
SGB V: Fünftes Sozialgesetzbuch (Fifth Social Code Book)
SHI: statutory health insurance.
